# Connective tissue nevus misdiagnosed as juvenile localized scleroderma

**DOI:** 10.1186/s12969-023-00913-9

**Published:** 2023-10-17

**Authors:** F. Tirelli, C. Giraudo, M. Soliani, F. Calabrese, G. Martini, P. Gisondi, A. Meneghel, Francesco Zulian

**Affiliations:** 1 Rheumatology Unit, Department of Woman and Child Health, University Hospital of Padova, Via Giustiniani 3, Padova, 35128 Italy; 2 Radiology Institute, Unit of Advanced Clinical and Translational Imaging, Department of Medicine-DIMED, University of Padova, Padova, Italy; 3 Pediatric Unit, ASST Cremona, Cremona, Italy; 4 Department of Cardiac, Thoracic, Vascular Sciences and Public Health, University of Padova, Padova, Italy; 5 Department of Medicine, Section of Dermatology and Venereology, University of Verona, Verona, Italy

**Keywords:** Scleroderma, Juvenile scleroderma, Morphea, Connective tissue nevus, Hamartoma, Pediatric dermatology

## Abstract

**Background:**

Connective tissue nevi (CTN) are congenital hamartomas caused by excessive proliferation of dermis components. In children, CTN can mimic juvenile localized scleroderma (JLS), an immune mediated skin disorder that requires aggressive immunosuppression. Objectives: Aim of our study was to describe a series of pediatric patients with CTN misdiagnosed as JLS and the discerning characteristics between the two conditions.

**Methods:**

Retrospective analysis of children referred to our Center during the last two decades for JLS who received a final diagnosis of CTN. Clinical, laboratory, histopathological and instrumental data (MRI and thermography) were collected and compared with those with JLS.

**Results:**

Seventeen patients with mean age at onset 4.6 years entered the study. All came to our Center with a certain diagnosis of JLS (n = 15) or suspected JLS (n = 2). The indurated skin lesions were flat and resembled either circumscribed morphea or pansclerotic morphea. In 14 patients (82.4%) they were mainly localized at the lower limbs and in three (17.6%) at the upper limbs. No patient had laboratory inflammatory changes or positive autoantibodies. Skin biopsies confirmed the diagnosis of CTN: non-familial collagenoma in eleven (64.7%), mixed CTN in four (23.5%) and familial CTN in two (11.8%). Mean age at final diagnosis was 9.5 years, with a mean diagnostic delay of 4.8 years (range 1–15 years). Sixteen patients underwent musculoskeletal MRI that was normal in all except two who showed muscle perifascial enhancement. Thermography was normal in all patients. At our first evaluation, eleven patients (64.7%) were on systemic treatment (methotrexate 11, corticosteroids 7, biologics 2), three (17.6%) on topical corticosteroids and three untreated.

**Conclusions:**

CTN can be misdiagnosed as JLS and therefore aggressively treated with prolonged and inappropriate immunosuppression. The absence of inflammatory appearance of the skin lesions, normal instrumental and laboratory findings and the accurate evaluation of skin biopsy are crucial to address the right diagnosis.

**Supplementary Information:**

The online version contains supplementary material available at 10.1186/s12969-023-00913-9.

## Introduction

Connective tissue nevi (CTN) are rare hamartomas of the dermis resulting from abnormal structure of the extracellular matrix component [1, 2]. They are usually classified according to which dermal component (collagen, elastin, or glycosaminoglycans) is found in excess and/or to the genetic patterns of inheritance [1–4]. Most reports are of sporadic lesions, but familial cases occur, suggesting an autosomal dominant transmission [3–5]. Moreover, CTN can manifest as isolated lesions or be part of systemic diseases such as Buschke-Ollendorff syndrome, which associates with collagenomas and elastomas, or tuberous sclerosis which has shagreen’s patches, another type of collagenoma, as associated feature [6]. Clinically, CTN appear as firm, asymptomatic, skin-coloured plaques often composed by adjacent papules with an orange peel–like surface texture, without significant dyspigmentation and with irregular and poorly defined borders located anywhere on the cutaneous surface of the body [1, 2]. Despite several differences in the presentation, some forms of CTN can mimic Juvenile Localized Scleroderma (JLS), the most common scleroderma subtype in childhood, which is a chronic skin disease characterized by inflammation and progressive fibrosis of the skin [7–9]. Discriminating between these two conditions is crucial, as JLS often requires aggressive immunosuppression [10–12], while CTN, being hamartomas, do not display inflammatory features and mostly benefit from an intense physiotherapy program to prevent deformities [7]. After evaluating a few patients who came under our observation for “atypical” or “treatment-refractory” juvenile localized scleroderma (JLS) and who were eventually diagnosed as CTN, we analyzed the cohort of patients with CTN, referred to our Center during the last two decades, to evaluate the clinical, laboratory, radiological and histopathological differences with classical JLS to avoid misdiagnosis and inappropriate immunosuppressive treatment.

## Patients and methods

Demographic data were collected by retrospective chart review of patients with CTN, diagnosed histologically according to the recent classification criteria [3, 4], evaluated between January 2001 and December 2021 at our Pediatric Rheumatology Unit, who came to our observation for “atypical” or “treatment refractory” JLS [7]. Data collected included: demographics (age, gender), clinical data (age at disease onset, age at diagnosis, delay in diagnosis, presence of antinuclear antibody (ANA) considered positive if ≥ 1:160 and/or extractable nuclear antigens antibody (ENA), clinical presentation (appearance and site of the lesion, lesion enlargement during the first years and functional disability), skin pathology results, instrumental evaluation (magnetic resonance imaging (MRI), infrared thermography (IRT)) and treatment history with topical (tCS) or systemic corticosteroids (sCS), methotrexate (MTX) and/or biological agents (BA). Disease activity was evaluated by using the Localized Scleroderma Assessment Tool (LoSCAT) [13] combined with infrared Thermography (IRT) [14]. LoSCAT is composed of two parts: the Localized Scleroderma Skin Severity Index (mLoSSI) that evaluates disease activity by grading of three domains: new lesion/lesion extension, erythema and skin thickness and the Localized Scleroderma Skin Damage Index (LoSDI) that is composed by three domains: dermal atrophy, subcutaneous atrophy and dyspigmentation. IRT examination was performed with an infrared camera (ThermaCAM PM695, FLIR systems AB, Stockholm, Sweden) at room temperature, after 20 min of acclimatization, wearing underwear. Lesions were considered positive to IRT when they were at least 0.5 °C warmer than the surrounding area or contralateral side.

Deep skin biopsies of the lesion were either performed at the referring Center and initially examined by a local pathologist, or performed at our Center. An expert dermatopathologist (FC) then reviewed the histological samples of all the included patients and provided the final diagnosis. The histopathologic criteria [3] for the diagnosis of CTN included thickened collagen bundles arranged randomly in the reticular dermis sometimes extending into the upper subcutis (collagenoma) and/or thick, branching, and interlacing elastic fibres (seen by elastic tissue staining) in the mid and reticular dermis (elastoma), without calcified or fragmented elastin fibres, inflammatory infiltrate, or adnexal changes suggestive of JLS. According to the most recent criteria [1–4], specimens were classified into four possible histopathologic groups: ‘‘pure’’collagenoma referring to collagen fibre changes only; ‘‘pure’’ elastoma, showing elastic fibre changes only; mixed type CTN with both collagen and elastic changes and cellular CTN, in which an increased number of normal-looking fibroblasts was present in addition to fibre changes [3, 4]. Musculoskeletal MRI images were all analysed by the same expert radiologist (CG). In selective cases, bone radiographs (limbs, pelvis) were also carried out to exclude Buschke-Ollendorff Syndrome (BOS) [5, 6]. All data obtained from CTN patients were compared to those observed in typical JLS patients [7, 8]. According to the Padua University Hospital policy, approval from the Ethics Committee was not needed because all data were anonymously collected.

## Case histories

As instructive examples, we briefly report the clinical histories of two patients (Table 1).

**Table 1:** Demographics and clinical characteristics of the patients

Patient	Gender	Age at Onset (y, m)	Age at Diagnosis (y, m)	Delay in Diagnosis (y, m)		Lesion Site	Initial Diagnosis	Previous therapy	First Pathology Result	Final Diagnosis
1	M	2	8.4	6.4	LL, T		JLS	MTX	JLS	Non-familial collagenoma
2	F	4	9	5	LL, T		JLS (suspected)	None	ND	Non-familial collagenoma
3	F	3	8.6	5.6	LL, T		JLS	tCS	Undefined	Non-familial collagenoma
4	M	9	13.4	4.4	UL		JLS	sCS, MTX	JLS	Mixed CTN
5	M	2	3.8	1.8	LL		JLS	tCS	JLS	Familial collagenoma
6	F	2	6	4	LL		JLS	tCS	JLS	Familial collagenoma
7	F	8	10	2	LL, T		JLS	sCS, MTX	ND	Non-familial collagenoma
8	F	5	6	1	UL		JLS	sCS, MTX	JLS	Non-familial collagenoma
9	F	9	11	2	LL		JLS	sCS, MTX, BA	JLS	Mixed CTN
10	M	2	8	6	LL, T		JLS	sCS, MTX	JLS	Mixed CTN
11	F	2	9	7	LL		JLS/Fasciitis	sCS,MTX	Undefined	Mixed CTN in BOS
12	F	2	6	4	UL		JLS (suspected)	None	ND	Non-familial collagenoma
13	F	7	11	4	LL		JLS	MTX	JLS	Non-familial collagenoma
14	M	4	10	6	UL, LL		JLS/Fasciitis	None	Fasciitis	Non-familial collagenoma
15	F	3	6	3	LL, T		JLS	sCS, MTX, BA	JLS	Non-familial collagenoma
16	M	8	13	5	LL		JLS	sCS, MTX	JLS	Non-familial collagenoma
17	F	6	21	15	LL		JLS	MTX	JLS	Non-familial collagenoma

Legends: LL: lower limbs; UL: upper limbs; T: Trunk; CTN: connective tissue nevus; JLS: juvenile localized scleroderma; BOS: Buschke Ollendorff Syndrome; CS: topical corticosteroids; sCS: systemic corticosteroids; MTX: methotrexate; BA: biological agents; ND: not done

Case no. 9 A previously healthy caucasian girl was referred to our Center when she was 11 with a history of progressing linear scleroderma refractory to prednisone (PDN), methotrexate (MTX), mofetil mycophenolate (MMF) and Tocilizumab (TCZ).

Two years earlier, the patient suffered from pain-discomfort in her left hip while playing handball. She was referred to a physical therapist who found leg length discrepancy (1.5 cm) as well as decreased range of motion of the left hip due to the tightness of the skin.

She was evaluated by a dermatologist who reported an indurated, firm feeling area over the lateral aspect of the upper left thigh. A skin biopsy revealed “focal compact collagen with no inflammation which could be consistent with morphea”. She was started on weekly methotrexate (MTX) 15 mg with mild improvement.

Six months later, she was evaluated at an academic pediatric rheumatology department for difficulty walking. Physical exam showed left buttock hard to touch with mild *peau d’orange* appearance. From the left hip, laterally down to the knee there was a sclerotic skin band about 7–10 cm wide with no hyperemic/hyperpigmented border. ANA and other scleroderma-specific autoantibodies were negative. The diagnosis of linear scleroderma was confirmed, therefore she continued methotrexate. Nine months later, an increased hardening of the skin around the knee caused a worsening of flexor contracture. The treatment was intensified to MTX 17.5 mg weekly with methylprednisolone (MPDN) pulses for three days (30 mg/kg, i.v.) followed by daily PDN at 0.8 mg/kg.

Two years after the symptoms start, new areas of induration over the left side by her ribs, lower leg and dorsum of foot were discovered. Physical exam was notable for a cushingoid face without striae and hair loss. The skin over the left flank felt tighter than the right one, the same for the left upper and lower leg laterally, as well as the left buttock. MRI: showed minimal induration of the subcutaneous adipose tissue laterally in the left proximal upper leg with mild thickening of the skin. No evidence for myositis or fasciitis. Pulmonary function tests (PFTs), including DLCO, were normal. A second skin biopsy revealed no abnormalities of the epidermis while dermis showed collagenous and partially hyalinated connective tissue with mucin deposition but no inflammatory cells, a picture consistent with morphea/atrophoderma of Pasini and Pierini. She was considered refractory to treatment, therefore she underwent three more pulse MPDN infusions followed by PDN 0.5 mg/kg daily and initiation MMF at the dose of 1000 mg/m2 daily. Six months later she was seeing at another Center and Tocilizumab i.v. (standard dose) every two weeks was added.

**Fig. 1:**
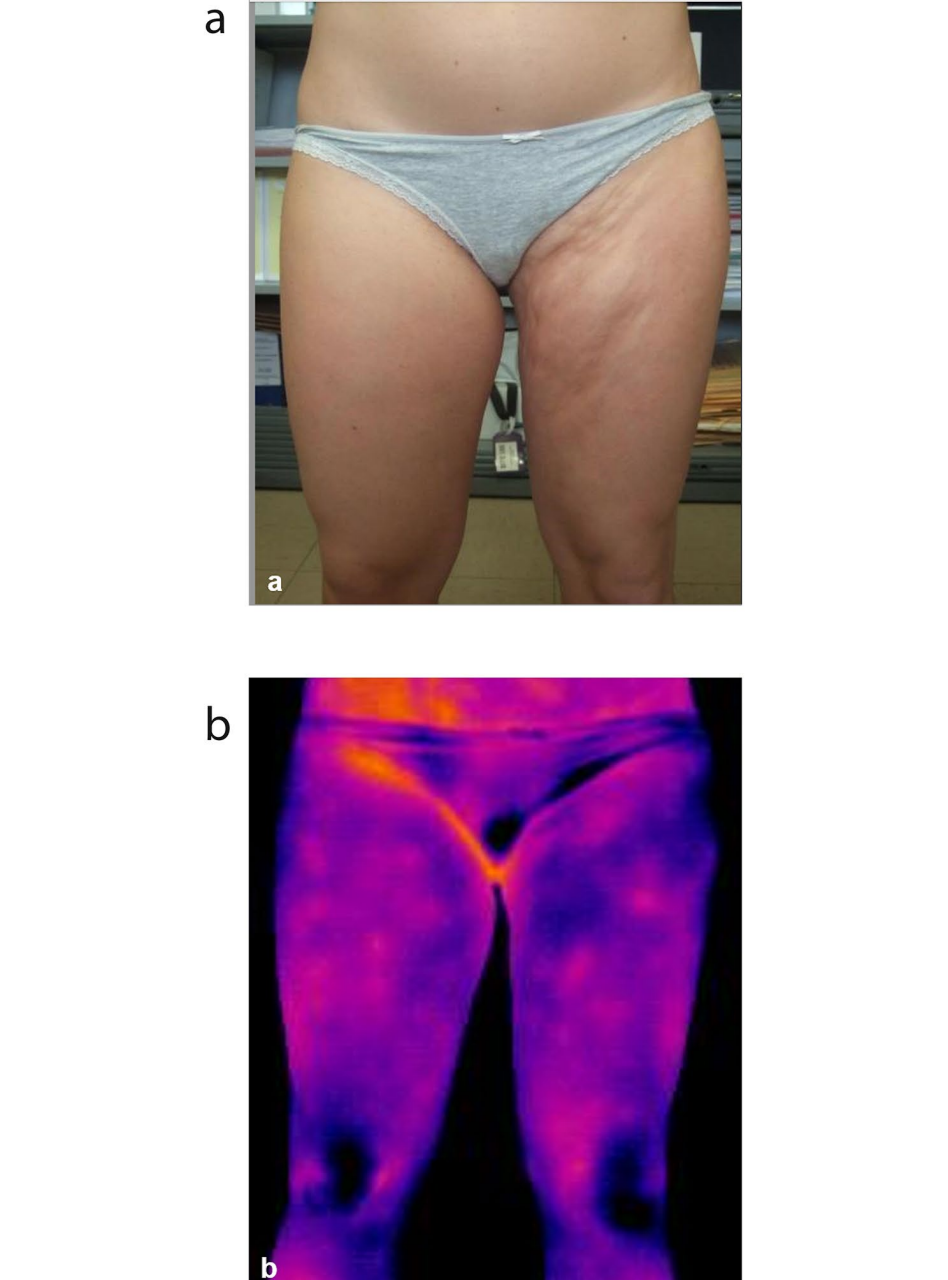
Clinical presentation of patient no. 2 at the age of 18. (**a**) Clinical appearance of the lower limb Collagenoma (**b**) Infrared Termography findings showing no sign of inflammation

When she came to our observation, physical examination showed a large indurated skin lesion that involved the entire left buttock and extended down to left thigh and hip, no epidermal changes nor lilac ring (Fig. 1A). The hard border of the lesion was palpable. Intra and extra rotation of the left hip were limited and the patient had a clear cushingoid appearance.

The physical examination with lack of hyperemic/hyperpigmented borders of the indurated lesions, the negative IRT (Fig. 1B) and the revision of the skin histology were suggestive of Connective Tissue Nevus (mixed subtype) [3]. She stopped the immunosuppressive treatment and started an intensive rehabilitation program in order to favor a normal limb motility and prevent deformities of the spine.

Case no. 15 A previously healthy caucasian girl was referred to our Center when she was 6 with a three years history of presumed “localized scleroderma” refractory to PDN, MTX and TCZ. Since birth, she presented, in the right periumbilical area, a small area of skin thickening. From three months to three years, she was followed by a dermatologist at an academic hospital but she did not undergo any treatment. When she was three, the parents noticed another area of skin thickening, about 2 cm in diameter, at the lateral region of the right thigh, initially interpreted as “panniculitis”.

At 5 years, following a new dermatological consultation, the suspicion of localized scleroderma was raised and blood tests including ANA, ANCA, ENA resulted negative or normal. The MRI of the thigh showed anterolateral thickening of the subcutaneous tissue of the proximal third of the right thigh, extending distally for about 5 cm. Capillaroscopy was normal. Chest CT, spirometry with DLCO, ECG and Echocardiography were normal. A skin biopsy showed characteristics “compatible with the diagnosis of scleroderma”, therefore, the patient was admitted into the hospital and treated with three daily pulses of MPDN (30 mg/kg), followed by daily PDN (1 mg/kg for 3 months), associated with weekly MTX 15 mg s.c. Three months later, given the lack of response to treatment, the patient underwent 10 monthly infusions of TCZ. At 6.2 years of age, the patient came to our observation. Physical examination revealed a large abdominal area of skin thickening with a *peau d’orange* appearance and ill-defined border, extending from the navel towards the right flank with no altered pigmentation nor atrophy; a thickened plaque with the same features extended laterally from the root to the distal right thigh. No functional limitations. Slight dorsal right-concave traction scoliosis. IRT showed no significant areas of hyperthermia. The clinical features, MRI and thermography were suggestive of Collagenoma [3]. The diagnosis was confirmed by the histological revision of the biopsy performed elsewhere, one year earlier. The patient then stopped the immunosuppressive therapy and underwent an intense rehabilitation program to counteract the traction scoliosis.

## Results

Seventeen patients (6 males, 11 females), mean age at onset 4.6 years (range 2–9 years), with suspected JLS seen at our Center in about two decades, entered the study. All patients came for a second opinion with a certain (n = 15) or suspected (n = 2) diagnosis of JLS made elsewhere. In the same period of time, we evaluated 153 patients with new onset JLS, therefore this group of 17 patients with CTN represents 11% of total. The clinical characteristics and the treatment at the time of our first evaluation are summarized in Table 1. In all patients the indurated lesions did not present areas of skin elevation, partially resembled circumscribed morphea and showed indefinite edges that could only be appreciated on palpation (Fig. 1A). In two, they involved most of a lower limb, partially resembling pansclerotic morphea [7]. In 14 patients (82.4%) the indurated skin lesion was localized at the lower limbs and in three (17.6%) at the upper limbs. In six patients (35.3%) lesions with the same characteristics were present on the trunk, too. No evident signs of skin inflammation were present.

At the time of our first observation, eleven patients (64.7%) were on systemic treatment (MTX 11, oral PDN 8, biologics 2), three (17.6%) on topical corticosteroids and only three were untreated (Table 1). Lesions were reported as moderately expanding during the first few years from onset in six patients. The mean age at onset in this group of children with expanding lesions (3.7 years) did not significantly differ from that of the remaining eleven (4.8 years) who showed modest or no progression (t-test, p = n.s.). Five out of six patients with progressive lesions came under our observation already on immunosuppressive systemic treatment, including two on biological agents (Tocilizumab). In four patients with CTN crossing the joints, flection contractures and mild disability were observed. In three children with trunk involvement, the CTN progression caused kyphoscoliotic deformities. Only one patient presented BOS [5]. This patient, other than a mixed collagen-elastin histological pattern, had the characteristic bone lesions of BOS that was confirmed by genetic testing.

As the referring diagnosis was JLS, the LoSCAT score was applied for clinical scoring. The activity score (LoSSI), part of the LoSCAT score, showed essentially grade 0–1 erythema and grade 1–2 skin induration in all patients. The application of the damage index (LoSDI) revealed that no CTN patient presented dermal or subcutaneous atrophy but only mild degree (grade 1–2) hyperpigmentation. Of interest, conversely to what we observe in JLS, neither LoSSI nor LoSDI changed over time.

**Fig. 2:**
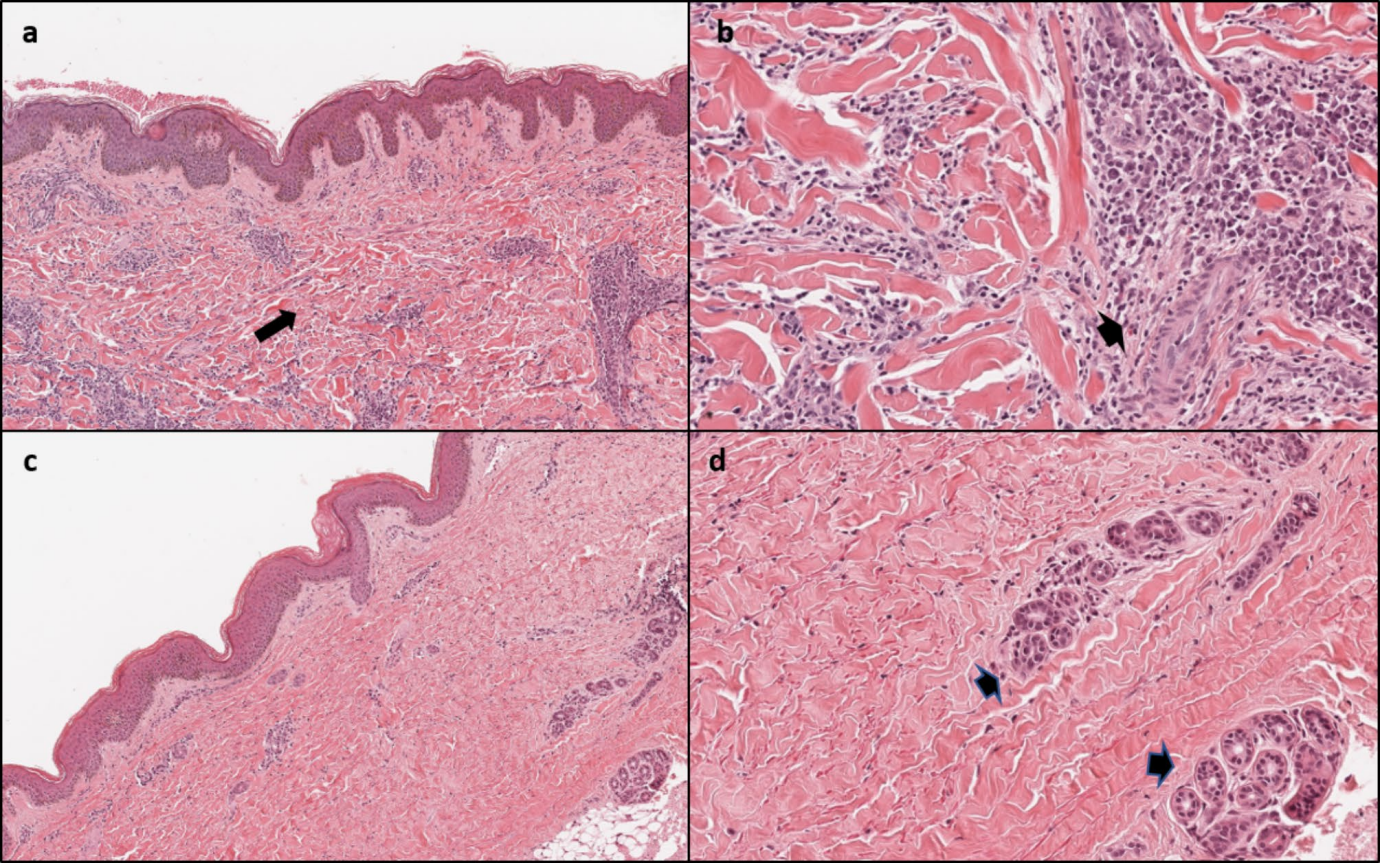
Comparison of histological characteristics of juvenile localized scleroderma and connective tissue nevus. Juvenile localized scleroderma: (**a**) compact fibrosis involving dermal and subcutaneous layers (long arrow), disappearance of skin adnexa, (**b**) perivascular inflammatory infiltrates (short arrow). Non-familial collagenoma: (**c**) thickened collagen bundles arranged randomly in the reticular dermis (long arrow), (**d**) preserved skin adnexa and absence of inflammatory infiltrate are evident (short arrow) *(Hematoxylin Eosin, original magnification a-c: 70x, b-d: 200x)*

We performed a deep skin biopsy in three children at our center, while 14 children had already undergone a skin biopsy at the referring center. The initial reported pathology was JLS in 11, fasciitis in one and undefined in two. Analysis of skin biopsies performed in our center and the histological review of the remaining 14 performed elsewhere confirmed the diagnosis of CTN in all (Table 1; Fig. 2A and B). The final diagnosis was non-familial collagenoma in eleven (64.7%), mixed (collagen-elastic) CTN in four (23.5%) and familial collagenoma in two (11.8%). Mean age at final diagnosis was 9.5 years, with a mean diagnostic delay of 4.8 years (range 1–15 years). Sixteen patients underwent a musculoskeletal MRI that was normal in all except three who showed mild subcutaneous atrophy in one (Pt. 2) and muscle perifascial enhancement in two (Pts. 4 and 16) (Table 1). IRT was normal in all patients (Fig. 1B).

No patient had elevation of the laboratory inflammatory parameters or positive autoantibodies.

## Discussion

In this work, we reviewed the clinical features of a series of pediatric patients with CTN and compared to those of children with JLS. Our results provide a set of clinical, laboratory and instrumental elements for the differential diagnosis and underline the importance of an early discrimination between the two clinical entities. First, our study clearly demonstrates how the incorrect diagnosis of CTN led to a significant diagnostic delay (mean 4.8 years, range 1–15 years). In some cases, this was due to an erroneous interpretation of the histological examination that led to the diagnosis of JLS. For this reason, at the time of our observation, two thirds of patients were being treated with systemic immunosuppressive drugs, usually recommended in JLS [12]. Two of them even tried biological agents, generally reserved to the most severe cases of JLS [15, 16], with the risk of potential side effects. One of the possible causes that led to a misdiagnosis of JLS was related to the progressive course in some of them, typically in the first years after the discovery of the lesion by the parents [3]. In fact, most of the patients who showed progression were already being treated with systemic immunomodulatory treatment at the time of our evaluation. Saussine et al. reported that monomelic forms of CTN affecting the lower limbs are more prone to expansion and therefore might be more often mistaken as JLS [3]. Indeed, according to these Authors, they typically affect younger children. In our series, we did not find significant difference between the age of the six children with progressive forms and the remaining eleven patients who showed modest or no progression. Indeed, as response to CS and MTX is usually very good in most cases of JLS [10, 11, 17], the absence of treatment response has raised the doubt of a different diagnosis. Our results suggest that several other clinical features can help address a correct differential diagnosis and thus avoid unnecessary treatments (Table 2).

**Table 2:** Main findings of connective tissue nevus as compared to juvenile localized scleroderma

		CTN*	JLS**
Clinical Appearance	*Borders*	Poorly- or undefined	Defined
	*Skin colour*	Normal	Hyperemic or hyper-hypopigmented
	*Skin adnexa*	Present/increased	Decreased or absent
Autoantibodies (%)	*Antinuclear antibodies (ANA)*	Negative	30–70
MRI Abnormalities (%)	*Subcutaneous atrophy*	5.9	70–90
	*Bone marrow edema*	Absent	5–36
	*Muscle edema*	Absent	15–71
	*Muscle fat replacement*	Absent	7.1
	*Sarcopenia*	Absent	57
	*Perifascial enhancement*	11.8	16–35
Pathology	*Collagen fibers*	Grossly thickened	Thickened
	*Skin adnexa*	Normally represented	Decreased/absent
	*Inflammatory infiltrate*	Absent	Perivascular, perineural

Legend: CTN connective tissue nevus; JLS juvenile localized scleroderma; ANA antinuclear antibodies; MRI Magnetic Resonance Imaging

*Present series

**Summary of references 10–12, 18–21

Generally, the gender distribution is similar in CTN and JLS, being the F:M ratio 2.2:1 and 2.4-4:1, respectively [3, 9, 18], while the age at onset is significantly lower in CTN (mean 2–4.4 years) [3] than JLS (mean 6.4–8.7 years) [9, 18].

In CTN, the clinical presentation varies widely based on the subtype [3, 4, 6] but some key feature can help in the differentiation from JLS. As shown in Fig. 1a, CTN usually present as plaques that tend to converge together, often with poorly demarcated edges that can be appreciated only by palpation. The skin presents a peel-like appearance with normal color, in contrast with the pearlescent aspect and dyspigmentation typical of JLS. Moreover, in CTN, skin adnexa are normally represented and the erythematous halo and other signs of inflammation, typical of active JLS, are absent. Moreover, also signs of damage, such as dermal and subcutaneous skin atrophy are not present. Indeed, according to this clinical features and conversely from what is seen in JLS [13], in our patients the LoSCAT scoring only showed absent or low grade of disease activity and in most cases no signs of disease damage, apart from mild hyperpigmentation. Also, lack of significant changes in the score over time, rather frequent in JLS, allows differentiating CTN from JLS.

Laboratory is also of some help as serum inflammatory markers and autoantibodies are normal or negative in CTN, while they are elevated or positive in 22–35% and 30–70% of JLS patients, respectively [9, 18].

Infrared thermography is a recognized valuable tool in the assessment and follow up of JLS as it identifies areas of cutaneous hyperthermia, which are related to disease activity, even not evident on inspection [14]. Once again, the absence of inflammatory features in CTN resulted in normal IRT results in all patients (Fig. 1b).

Muscle skeletal MRI provides useful insights in the definition of JLS extension, especially in the characterization of deep tissue involvement [19–21]. As summarized in Table 2, MRI findings of CTN significantly differ from those reported in JLS, especially regarding the deep tissue involvement [19]. Finally, CTN pathology was the key element that eventually allowed a correct diagnosis in all patients. According to the recent criteria [3, 4], the presence of grossly thickened collagen fibers, the normal representation of vascular structures and skin adnexa, together with the absence of significant perivascular and perineural inflammatory infiltrate are the most important features to differentiate JLS (Fig. 2a and b) from CTN (Fig. 2c and d).

As a limitation, other than the retrospective nature of this descriptive case series, we acknowledge that of the tools described, such as IRT, might not be universally available for pediatric dermatologists and rheumatologists. Nonetheless, awareness about CTN is important when evaluating a thickened skin lesion suspect for scleroderma, as the relevant differences upon clinical examination can guide the clinician to initiate the differential diagnostic work – up and possibly refer the patient to a specialized center. Lastly, as underlined by the case of the patient in our series who received a final diagnosis of BOS, a suspicion of CTN can also lead to perform a full examination and collect clinical data to rule out possible associated systemic diseases.

## Conclusion

In our experience, CTN is the most frequent condition to be included in the differential diagnosis with JLS. Pediatric rheumatologists and dermatologists should be aware about this condition in order to avoid inappropriate and prolonged immunosuppressive treatments. In doubtful cases, a deep skin biopsy is mandatory to address the right diagnosis.

### Electronic supplementary material

Below is the link to the electronic supplementary material.

Supplementary Material 1

Supplementary Material 2

## Data Availability

The datasets generated and/or analyzed during the current study are not publicly available (contains patients information).

## Abbreviations

ANA: Antinuclear antibodies
CTN: Connective tissue nevus
F: Female
JLS: Juvenile localized scleroderma
M: Male
