# Supplementary material for: Connective tissue nevus misdiagnosed as juvenile localized scleroderma
Source: Pediatr Rheumatol Online J. 2023 Oct 17;21:125. doi: 10.1186/s12969-023-00913-9 (PMC10583392; doi:10.1186/s12969-023-00913-9)
Supplement: Supplementary file 2 — Supplementary Material 2 [file 12969_2023_913_MOESM2_ESM.doc]

Prof Alberto Martini,
Prof Charles Spencer,

Editors *of Pediatric Rheumatology*

October 8, 2023

Dear Editors,

Please find enclosed the revised version of the manuscript entitled:

***“******Connective tissue nevus misdiagnosed as juvenile localized scleroderma”***

that we would like you to consider for publication in *Pediatric* *Rheumatology* as *Research Article*.

All reviewers’ comments have been addressed and the text has been changed if needed.

All Co-Authors agree on this new version and certify that the material contained herein has not been submitted or published elsewhere

With best regards,

Sincerely,

Francesco Zulian, M.D.
